# New 5-Modified Pyrimidine Nucleoside Inhibitors of Mycobacterial Growth

**Published:** 2010-04

**Authors:** L.A. Alexandrova, E.R. Shmalenyuk, S.N. Kochetkov, V.V. Erokhin, T.G. Smirnova, S.N. Andreevskaia, L.N. Chernousova

**Affiliations:** Engelhardt Institute of Molecular Biology, Russian Academy of Sciences; Central Tuberculosis Research Institute, Russian Academy of Medical Sciences

**Keywords:** tuberculosis, mycobacterium tuberculosis, anti-TB drug, nucleoside, 5-substituted pyrimidine, inhibitor

## Abstract

The WHO has declared tuberculosis (TB) a global health emergency. Therefore, there is an urgent need to discover and develop new anti–TB drugs. Here we report on a new category of 5–substituted pyrimidine nucleosides as potent inhibitors of Myco–bacterium tuberculosis growth in vitro. A series of 2ʹ–deoxy–, 3ʹ–azido–2ʹ,3ʹ–dideoxy–, and 3ʹ–amino–2ʹ,3ʹ–dideoxypyrimidine nucleoside analogues bearing lengthy flexible alkyloxymethyl substituents exhibited marked inhibitory activity against M. tuberculosis in vitro. 5–Dodecyloxymethyl–2ʹ–deoxyuridine was found to be a potent inhibitor of M. tuberculosis propagation in vitro. In contrast, monophosphates of the tested nucleosides were devoid of antimycobacterial activity. This new class of inhibitors seems to be a promising chemotherapeutic agent against TB and merits further studies.

## INTRODUCTION

Tuberculosis (TB) is one of the largest problems facing modern health services. At the beginning of the 21st century, TB is one of the most widespread infectious diseases: about one–third of the world’s population (more than two billion people) is currently infected with *Mycobacterium tuberculosis*. According to WHO reports, about nine million people are infected every year, and more than two million people, of whom 10% are also infected with HIV, die from tuberculosis [1, 2]. Infection with HIV increases the chance of latent TB reactivation [3] or leads to rapid TB development soon after (re)infection with the TB bacillus [4]. Latent–to–active TB transition risk reaches 50% among people with AIDS and is about 10% among the rest of the population.

Effective and accessible TB treatment schemes based on combinations of different drugs were developed at the beginning of the 1950s. More than ten anti–TB drugs may be included in the chemotherapy scheme [5]. Since this time, the broad use of anti–TB drugs, as well as vaccination, has led to a significant decrease in TB–related mortality. On the other hand, the drug usage induced the selection of strains resistant to several pharmaceuticals of various types. HIV infected, drug–dependent, and transplant patients have extremely weakened immunities and, hence, become TB victims. These circumstances led to the fact that the frequency of TB cases started to grow at the end of the 1980s. The WHO announced a global TB health emergency in 1993. New *Mycobacterium tuberculosis* strains should be particularly noted: multidrug and extensively drug–resistant tuberculosis (M/XDR–TB) strains [1] that are barely affected by standard chemotherapy schemes. M/XDR–TB generally results from incorrect treatment, when patients receive insufficient amounts of anti–TB drugs [6]. Usually, it takes more than half a year from the beginning of anti–TB treatment until the patient recovers, and during this time any contact with an infected patient can result in contamination [5]. The main factor determining resistance development under the action of anti–TB pharmaceuticals is the selection of drug–resistant mycobacteria with genome mutations.

TB is one of the largest problems in Russia, because its current incidence rate is 190.5, prevalence is 85.1, and mortality rate is 17.9 cases per 100 000 people. At the same time, the occurrence of MDR–TB is 13.6% in the patients with new TB cases and 28.8% in patients with relapses, according to the statistics of the Ministry of Health and Social Development of the Russian Federation [7]. In connection with the above, the search for new anti–TB drugs is necessary.

Therapy for viral infections is frequently based on using natural nucleoside derivatives [8]. The anti–TB activity of nucleosides has not been revealed until recently. Recent reports have appeared on several groups of modified nucleosides displaying a remarkable anti–mycobacterial effect in experimental models [9–14].

Recently, 5–modified pyrimidine nucleosides with lengthy 1–alkinyl radicals have demonstrated an inhibitory effect on *Mycobacterium tuberculosis* and *M. bovis in vitro* [11–14]. The best antibacterial activity has been demonstrated for nucleoside 5–(1–dodecynyl) and 5–(1–tetradecynyl) derivatives. The study on the influence of the carbohydrate fragment modification on the antibacterial properties of 5–modificed nucleosides has demonstrated that virtually all 2ʹ–deoxy–, 2ʹ, 3ʹ–dideoxy–, 3ʹ–fluoro–2ʹ, 3ʹ–dideoxy–, and 2ʹ–fluoro–2ʹ, 3ʹ–dideoxynucleosides, as well as acyclic and arabinonucleosides with long 1–alkynyl radicals, have displayed anti–TB activity [11–14]. This work is devoted to an investigation of the * Mycobacterium tuberculosis * growth inhibitory capability of newly synthesized 2ʹ–deoxy–, 3ʹ–azido–2ʹ, 3ʹ–dideoxy–, and 3ʹ–amino–2ʹ, 3ʹ–dideoxy–pyrimidine nucleosides containing lengthy alkyloxymethyl radicals at their position 5.

## MATERIALS AND METHODS

**Fig. 1:**
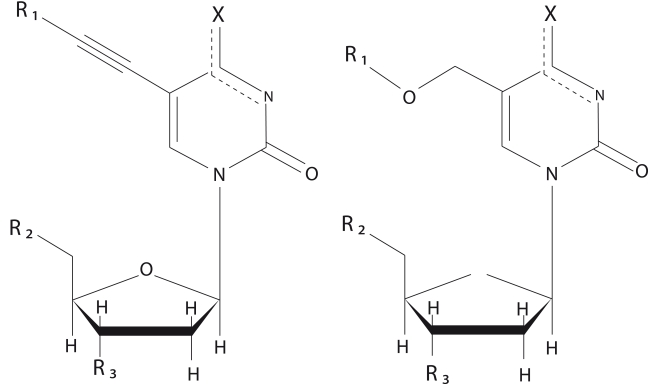
Structures of tested substances

**Tested substances** (Fig. 1, *1–10*). 5–(1–Dodecynyl)– and 5–(1–tetradecynyl)–2ʹ–deoxyuridine (*1* and *2*, respectively) used as a control were prepared by the method in [11]. 5–Alkyloxymethyl–pyrimidine nucleoside derivatives (*6–10*) were synthesized using our method in [15], and nucleoside 5ʹ–monophosphates (*3–5*) were synthesized by the method in [15–16].

**Mycobacterial strain**. These substances were tested using the *Mycobacterium tuberculosis* H37Rv laboratory strain sensitive to anti–TB drugs. A suspension of individual mycobacterial cells was equalized by the growth phase and standardized by CFU [17]. Cells were grown in a liquid medium Dubos with 5% BSA (Difco).

**Estimation of antibacterial effect of tested substances**. The effect that these substances have on the growth of the mycobacterial strain was examined using a BACTEC™ MGIT™ 960 Mycobacterial Detection System (BD, United States) for 24 days. Mycobacterium cell suspension (500 μL) was inoculated into 7.9 mL liquid medium 7H9 Middlebrook supplemented with OADC. The final concentration of *M. tuberculosis* in the sample was 10^5^–10^6^ CFU/mL. Each of the experiments with different tested substance concentrations, including control samples without any drug, was triplicated.

The antimycobacterial effect of the tested substances was estimated from the growth kinetics of * M. tuberculosis * H37Rv in the presence of a varied concentration of the tested substances compared with that in the absence of any drug [17]. Growth was monitored automatically every hour and recorded using Epicenter software (BD, United States). The mycobacterial growth was expressed in reference fluorescence units (RFU).

## RESULTS AND DISCUSSION

We studied the inhibitory effect of synthesized 2ʹ–deoxynucleoside derivatives (Fig. 1, *6–10*) carrying a long linear alkyl moiety introduced at position 5 of pyrimidine base via a oxymethyl group providing a higher flexibility of the hydrocarbon chain than 1–alkynyl derivatives (*1* and *2*) described in the literature [11–14] on the growth of *M. tuberculosis *. The method of pyrimidine nucleoside methoxyalkyl derivative synthesis we have developed [15] is essentially easier and cheaper than the proposed method of 5–(1–alkynyl)–nucleoside synthesis [11–14]. To reveal the role of 3ʹ–modification of the carbohydrate moiety in the anti–TB activity of 5–modified nucleosides, we have synthesized the following 2ʹ–deoxyuridine derivatives with the same substituent at position 5 of the base: 2ʹ–deoxy–, 3ʹ–azido–2ʹ, 3ʹ–dideoxy–, and 3ʹ–amino–2ʹ, 3ʹ–dideoxy–5–dodecyloxymethyluridine (Fig. 1, *8–10*).

**Fig. 2:**
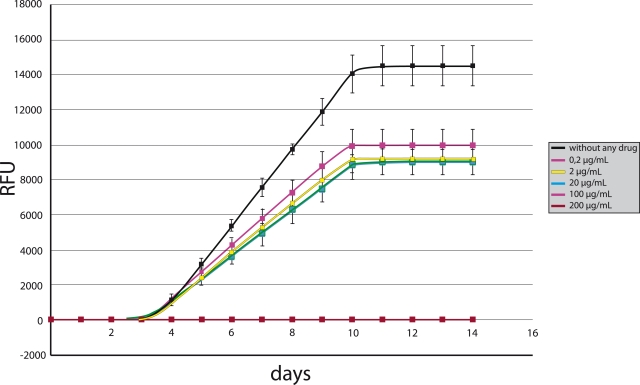
Experiment on testing the antimycobacterial activity of substances. The growth kinetic plot is drawn for the M. *tuberculosis* H37Rv culture exposed to a varied concentrations of substance 9. RFU is the reference fluorescence unit monitored using the Epicenter software (BD, United States)

The data on the bacteriostatic activity of the studied substances from the growth of mycobacteria in the automated Bactec MGIT960 system demonstrated that the *M. tuberculosis* H37Rv culture began to grow after 3.59 days in a medium without any drugs. The growth curve had a classic sigmoid shape with three phases: latent growth (before 3.59 days), exponential (log–phase or phase of active mycobacterial cell division) from 3.59 to 10.25 days, and a stationary one from 10.25 days until the end of the experiment (Fig. 2). The duration of the active cell replication phase was 6.66 days.

To confirm the antimycobacterial activity of nucleosides and adjust the experimental conditions, we primarily tested 5–(1–dodecynyl) and 5–(1–tetradecynyl)–2ʹ–deoxyuridine (*1* and *2*) at concentrations of 2, 20, and 200 μg/mL. According to published data [11], compounds *1* and *2* inhibited the growth of both *M. bovis *(MIC_90_ = 50 and 10 μg/mL, respectively) and *M. avium* by 50–70% at high concentrations. We showed that both substances taken at a concentration of 200 μg/mL completely inhibited the growth of *M. tuberculosis* culture.

5–Alkyloxymethyl derivatives of pyrimidine nucleosides (*6, 7, 9,* and *10*) and nucleoside monophosphates (*3–5***)** were tested at concentrations of 0.2, 2, 20, 100, and 200 μg/mL, and 5–dodecyloxymethyl–2ʹ–deoxyuridine (*8*) was tested at concentrations of 0.2, 2, 20, 50, and 100 μg/mL. The data of an experiment on determining the antimycobacterial activity is shown in Fig. 2.

A determination of the inhibitory effect of the substances *6–10 *on the culture of *M. tuberculosis* H37Rv has shown that substance *6* led to 100% growth inhibition at concentrations of 200 and 100 μg/mL, *7* at concentrations of 200 and 100 μg/mL, *8* at concentrations of 100 and 50 μg/mL, *9* at concentrations of 200 and 100 μg/mL, and *10* at concentrations of 200 and 100 μg/mL (Fig. 2). Thus, the minimal inhibitory concentration (MIC) is 100 μg/mL for the substances * 6, 7, 9, * and *10* and 50 μg/mL for the substance *8*.

Nucleosides with large hydrophobic groups at position 5 of the nucleic base are hardly soluble in water. To increase the solubility, we synthesized nucleoside 5ʹ–monophosphates (*3–5*) by the method in [16]; however, these substances do not inhibit the growth of mycobacteria even at high (200 μg/mL) concentrations, and the growth kinetics in the presence of these substances is the same as in the control (in the absence of any drug).

**Fig. 3:**
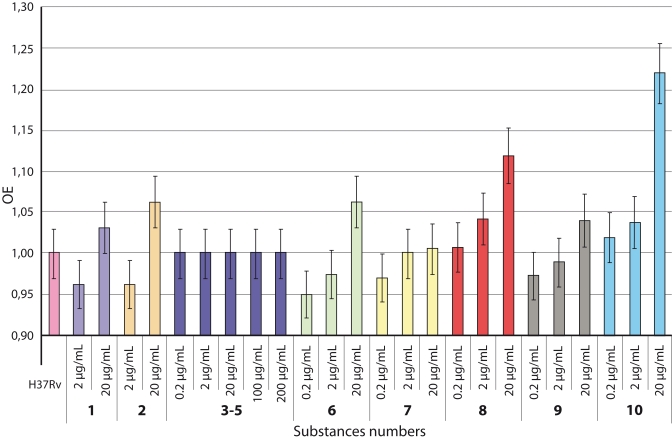
The efficacy of the substances inhibiting the M. *tuberculosis* culture growth at different concentrations. RE is the ratios between the active replication phase durations of the M. tuberculosis H37Rv culture exposed to the tested substances and that of the control culture, which was taken to be unity

We evaluated the active growth duration in the culture exposed to tested substances taken at concentrations not inducing 100% inhibition of the culture growth in comparison with the control (Fig. 3). The data is given in relative units (RU) calculated as the ratio of the active replication time of the culture growing in the presence of a substance to that of the control culture (*M. tuberculosis* H37Rv without any substance added). Fig. 3 shows that a more prolonged active replication phase is observed under exposition with 5–dodecyloxymethyl –2ʹ–deoxyuridine (*8*) and 5–dodecyloxymethyl–3ʹ–amino–2ʹ,3ʹ–dideoxyuridine (*10*) taken at a concentration of 20 μg/mL, which is significantly different from the control (p < 0.01) and suggests a decrease in the intensity of mycobacterial–cell replication in the culture. Note that 24 and 18 h delays (for the substances *8* and *10*, respectively) of mycobacterial growth when compared with the control were observed for these substances.

## CONCLUSIONS

The inhibitory effect that 2ʹ–deoxyuridine and 2ʹ–deoxycytidine derivatives with lengthy alkyloxymethyl moieties have on the growth of the *M. tuberculosis* H37Rv culture was first demonstrated in this work. 5–Dodecyloxymethyl –2ʹ–deoxyuridine (*8*) can be regarded as the most active against *M. tuberculosis* because of the lowest MIC of all tested compounds (50 μg/mL) and the most prominent capability of prolonging active cell replication and delaying the growth initiation at concentrations that do not cause 100% inhibition. Nonetheless, we have not revealed any fundamental difference in the inhibitory effect of pyrimidines differing both in the structure of the substituent (1–alkynyl or alkyloxymethyl moiety) at position 5 of the base and the carbohydrate fragment (2–deoxy–, 3–azido–2, 3–dideoxy–, or 3–amino– 2, 3–dideoxyribofuranose).

Thus, we have shown the capability of pyrimidine 2ʹ–deoxynucleoside 5–methyloxyalkyl derivatives to inhibit the growth of *M. tuberculosis in vitro*; the most effective derivatives could serve as prototypes for the development of new anti–TB drugs.

## ABBREVIATIONS

TB: tuberculosis
HIV: human immunodeficiency virus
MDR: multi-drug resistance
MIC: minimal inhibitory concentration
CFU: colony-forming unit
